# “It would be so much easier”: health system-led genetic risk notification—feasibility and acceptability of cascade screening in an integrated system

**DOI:** 10.1007/s12687-019-00412-z

**Published:** 2019-03-06

**Authors:** Nora B. Henrikson, Paula R. Blasi, Stephanie M. Fullerton, Jane Grafton, Kathleen A. Leppig, Gail P. Jarvik, Eric B. Larson

**Affiliations:** 1 Kaiser Permanente Washington Health Research Institute, Seattle, WA USA; 2 Department of Bioethics and Humanities, School of Medicine, University of Washington, Seattle, WA USA; 3 Kaiser Permanente Washington, Seattle, WA USA; 4 Departments of Medicine (Medical Genetics) and Genome Sciences, University of Washington, Seattle, WA USA

**Keywords:** Genetic testing, Cascade testing, Risk notification, Bioethics, Communication

## Abstract

Assess the feasibility and acceptability of health system-led genetic risk notification in a US integrated health system. We conducted semi-structured phone interviews with individuals age 40–64 years who had undergone genetic sequencing, but had not yet received their results, assessing attitudes to direct outreach to relatives. During each interview, we collected contact information for adult relatives identified as members of the same system and attempted to identify each relative in administrative data. We conducted 20 interviews. Most participants expressed support for Kaiser Permanente Washington involvement in familial risk notification. Direct outreach to relatives received the most unqualified support; outreach to the relatives’ physician or interaction with the relatives’ electronic medical record received more tempered support. Support was motivated by the desire to have risk communicated accurately and quickly. The most common caveat was a desire to alert relatives before the health system contacted them. Of 57 named relatives who were members of the same health system, we retrieved a single match for 40 (70.2%) based on name or birthdate. Health system involvement in familial risk notification received support in a sample of patients in a US integrated health system, and identification of relatives is feasible.

The American College of Medical Genetics and Genomics (ACMG) has identified a minimum list of 59 genes that laboratories should analyze for constitutional pathogenic or likely pathogenic variants whenever clinical genomic (exome or whole genome) sequencing is ordered because of the clinical utility, typically enhanced surveillance or clinical intervention, afforded by awareness of constitutional disease risk (Green et al. 2013). Such testing has immediate implications for the biological relatives of a tested person: parents, siblings, children, and other first-degree relatives may share the same gene variant and should accordingly be referred for genetic testing. However, uptake of testing among eligible relatives in the USA remains low: 20–30% for BRCA testing (Samimi et al. 2017) and slightly more for Lynch syndrome (Sharaf et al. 2013)

Currently in the USA, a person with an actionable pathogenic variant is responsible for contacting their own family members and communicating familial risk. Each family member then assumes responsibility for initiating genetic testing or follow-up care. However, up to a third of at-risk relatives may go unnotified, and thus miss the chance to benefit from genetic counseling, testing, and appropriate clinical follow-up (Taber et al. 2015; Graves et al. 2014; Fehniger et al. 2013). Because they insure and provide care for multiple generations of families, integrated health systems in the USA could begin cascade screening programs, in which relatives are systematically identified and invited for testing and follow-up. Such approaches have been implemented in other countries with favorable improvements in the uptake of genetic testing among relatives (Forbes Shepherd et al. 2017; Watts et al. 2011), but there has been little exploration of such approaches in the USA.

The aim of this study was to assess the feasibility and acceptability of health system–led identification of actionable pathogenic variants from next generation sequencing and risk notification of family members who receive care within the same health system.

## Materials and methods

We conducted semi-structured phone interviews with individuals who had undergone genetic testing using the eMERGEseq panel that included sequencing of 58 of the 59 ACMG recommended genes and other content, but had not yet received their results. For the feasibility aim of the project, we attempted to identify at-risk relatives in Kaiser Permanente Washington (KPWA) administrative data using only contact information provided by the proband. All study activities were approved by the KPWA Human Research Protection Program IRB.

### Population

The study population was a subsample of research participants enrolled in the KPWA/University of Washington (UW) phase 3 of the eMERGE study (eMERGE3), one of an ongoing consortium of sites studying the return of actionable genomic sequencing results in clinical systems. Participants for eMERGE3 were recruited into the Northwest Institute for Genomic Medicine Biorepository (NWIGM), and selected for personal history of colorectal cancer, colorectal polyps, or diverse ancestry. All participants were KPWA members recruited between 2008 and 2017.

The biorepository manages a collection of DNA samples for KPWA (formerly Group Health Cooperative) members with phenotyping accessible via electronic medical records. At the time of the study, all participants had agreed to receive genetic test results for all actionable pathogenetic and likely pathogenetic variants from the eMERGEseq panel (Fossey et al. 2018), but had not yet received their sequencing results. The time interval between enrollment in the interview study and having their samples sequenced ranged from 10 to 20 weeks; interview timing did not delay return of results for any participant.

### Sampling and recruitment

We identified a stratified random sample of participants at the eMERGE3 UW/KPWA site. Stratification was by gender and personal history of CRC or colorectal polyps. Eligibility criteria for participation included age 40–65 (to increase the likelihood of identifying a wider age range of relatives), reporting at least one relative who was also a member of KPWA (formerly Group Health Cooperative), and willingness to provide all KPWA relatives’ contact information. All participants provided written informed consent, and received $50 after the interview as a thank you. We mailed 100 invitations to participate, including a consent form and return postage and a number to call to schedule an interview or decline to participate. We reached out by phone to potential participants who did not call in.

### Interview topics

Interviews had two main topics (Table 1). First, we followed a use-case approach and designed two vignettes describing hypothetical return of results scenarios based on genetic conditions included in the ACMG list (Green et al. 2013), Marfan Syndrome (*FBN1*), and malignant hyperthermia (*RYR1*) (Table 2). We chose these two cases to describe varying levels of clinical urgency and complexity associated with the findings. Further, we sought to avoid a cancer risk variant since participants were awaiting sequencing results involving colorectal cancer genes and since some participants had a personal history of colorectal cancer. For each vignette, we asked participants which family members they would or would not share with, and what tools would help them share their results. We also asked about level of comfort with three different levels of KPWA involvement in notifying relatives who were also KPWA members: (1) KPWA contacting their family member directly, (2) KPWA contacting their family member’s KPWA physician, and (3) KPWA placing a note in their relatives’ medical record recommending genetic testing. We were intentionally vague on specific processes by which each of these might happen, partly because such processes do not yet exist and partly to retain a focus on an initial assessment of acceptability.

**Table 1:** Clinical vignettes

Vignette 1: Marfan syndrome (*FBN1*)	
Imagine that an eMERGE study doctor meets with you and explains that you have a variant in the *FBN1* gene. This variant is related to risk of a disease called Marfan syndrome. People with Marfan syndrome tend to be tall and have long thin fingers and toes. They also may have scoliosis (a curved spine), an indentation or protrusion of their breast bone, or eye problems such as being nearsighted. The biggest health concern for people with Marfan syndrome is that the aorta, the large blood vessel that carries oxygen through the body, becomes too wide. If the aorta becomes too wide, it can rupture and lead to sudden death. The study doctor explains that there is a lot we can do to help. With appropriate care and surveillance, people with Marfan syndrome can expect to live long productive lives. We would look at your heart regularly using an echocardiogram (ultrasound for the heart). We may suggest medications to keep the aorta from widening. If the aorta becomes too wide, it may require surgery. We may recommend avoiding activities such as contact sports or weight lifting. Children who have Marfan syndrome can start seeing a specialist for monitoring of their heart. Most people with Marfan syndrome inherit the genetic change from a parent. Other close relatives may have this same genetic change. The doctor explains that your family members may wish to be tested to discover if they too have inherited the same risk factor.	
Vignette 2: malignant hyperthermia (*RYR1*)	
Imagine that the eMERGE study doctor meets with you and explains instead that you have a variant in the *RYR1* gene. People with this variant are at risk for a condition called malignant hyperthermia, an abnormal reaction to certain medications used for general anesthesia during surgery. When they receive these medications, people with malignant hyperthermia have very high temperature, a fast heart rate, and breathing rate, and breakdown of skeletal muscles. They may die during surgery if not treated immediately. However, when we know that a person has this variant, we can let other doctors know to use different medications during surgery. People with *RYR1* alterations may also be at increased risk of heat stroke, and may need to avoid excess activity in high heat. If you have this variant, Kaiser Permanente would put an alert in your medical record so that if you ever needed surgery the team would know right away to avoid certain medications. The study doctor explains that it is likely that you inherited this variant from one of your parents. If you have brothers, sisters, or children, they might also wish to be tested to see if they have inherited the same risk factor.

**Table 2:** Participant demographics

	Interview participants *N* = 20	Overall sample *N* = 100
Female^a^	16 (80%)	58 (58%)
Age, mean (min, max)^a^	58.7 (41, 64)	61.6 (40, 64)
Race/ethnicity^a^		
White non-Hispanic	13 (65%)	72 (72%)
White Hispanic	2 (10%)	4 (4%)
Asian	5 (25%)	20 (20%)
Black/African American	0 (0%)	2 (2%)
Native American/Alaskan	0 (0%)	2 (2%)
Education^b^		
High school	2 (10%)	–
Some college	4 (20%)	–
Bachelor’s degree	9 (45%)	–
Post-graduate	5 (25%)	–
Employment^b^		
Employed full time	7 (35%)	–
Employed part time	5 (25%)	–
Self-employed	1 (5%)	–
Retired	7 (35%)	–
Marital status^a^		
Now married/living as married	15 (75%)	79 (79%)
Never married	3 (15%)	12 (12%)
Divorced	2 (10%)	9 (9%)
Income^a^		
$25,001–$50,000	7 (35%)	32 (32%)
$50,001–$75,000	6 (30%)	39 (39%)
$75,001–$100,000	1 (5%)	11 (11%)
more than $100,000	6 (30%)	18 (18%)
CRC status^a^		
None	10 (50%)	48 (48%)
Polyp	6 (30%)	25 (25%)
CRC dx	4 (20%)	27 (27%)

^a^From parent study data

^b^Assessed during semi-structured interview

For each case, we made clear that the patient would have to give their explicit permission, in accordance with Health Insurance Portability and Accountability Act (HIPAA) privacy rule (Rothstein 2018). In addition, we asked about any special considerations for relatives who are minors. We also asked a series of more general questions, focused on family closeness and frequency of interaction, how health information is shared within the family, and demographics. All interviews were conducted during 2017, recorded, and transcribed.

Second, we asked participants to identify all first- and second-degree relatives and to indicate which were current or former KPWA members. For each relative indicated as a current or former KPWA member, we collected all contact information that the participant had available, including name, address, birthdate, and address. We did not collect this information for current/former KPWA members who were under age 18.

### Analysis

#### Interviews

For interview data, we used a template analysis approach (Brooks et al. 2015). We created a set of analysis templates based on the interview guide questions and our study aims. After a pilot period using a subset of transcripts, we added emerging themes to the template as needed. We then completed the coding for the full set of interviews, with illustrative quotes. Templates were completed by one team member, with secondary coding by a second team member. We used content analysis to summarize attitudes toward each of the three hypothetical KPWA levels of involvement in results sharing for each of the two vignettes, based broadly on the level of support that was indicated for each, along with the caveats or concerns stated and exemplar quotes. Analyses were completed using Microsoft Excel.

#### Identifying relatives from contact information

Using KPWA administrative data, we sought to identify each relative named during the interviews. We used internal (KPWHRI) warehouse consumer files for name and address information, and the HCSRN VDW demographics table for gender and birthdate information.

First, we counted the number of matches for eight contact information fields for each relative. Then, we constructed cumulative sets of matches (in this order: last name, first name, birth year, birth month, birth day, gender, city, and ZIP code). We then evaluated the number of matches for each level, with a focus on the amount of information that would provide a single match. For those that did not match on both last and first name, we attempted to match on birthdate, and both last name and birthdate. In accordance with the feasibility design, as well as the terms of our IRB approval, we did not contact family members or conduct any kind of validation of identity. Therefore, we considered a match of a single person, with no duplicate matches, to be a successful match.

## Results

**Fig. 1:**
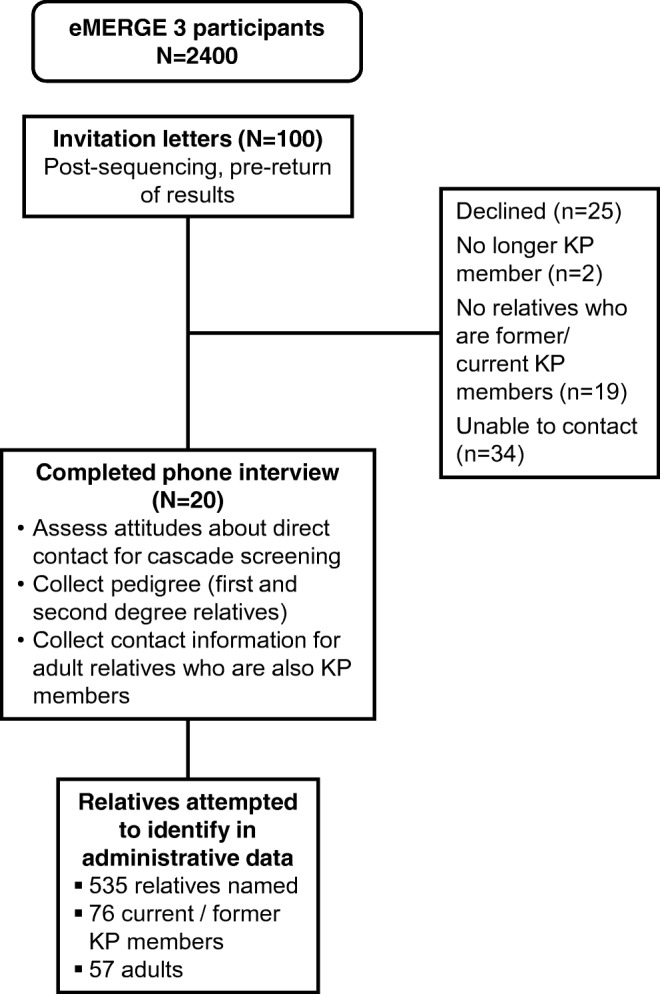
Study design flowchart

Of 100 mailed invitations, 35 people called the study team to discuss participating. Of the 35, 5 declined participation after discussing the study with project staff; 15 were ineligible because they did not have relatives with KPWA coverage; 1 was ineligible because they were no longer a KPWA member, and 14 consented to be interviewed (Fig. 1).

In addition, we attempted to call the 65 eMERGE participants who did not respond to the initial letter, and we reached 31 of the 65 participants by phone to discuss enrollment. Of the 31, 20 declined participation after discussing the study with project staff; 4 were ineligible because they did not have relatives with KPWA coverage; 1 was ineligible because they were no longer a KPWA member, and 6 consented to be interviewed.

The sample was self-identified as predominantly white or Asian; female, married, and college educated (Table 2). Interview participants were more likely to be female (80%) than the sample of 100 from which they were drawn (50%), but had similar marital status and income, and race/ethnicity to the larger sample. All reported being either employed or retired. Half (50%) were designated per their eMERGE participation as having had either CRC or colorectal polyps previously. Only one participant reported having children under age 18 living in the home.

### Family structure and health information sharing

Most participants reported close family relationships with all or some of their biological relatives. One participant stated that they were not very close to anyone in their family. Many people noted varying levels of closeness with different relatives depending on geographic distance or relationship factors. In-person or phone communications were the most commonly reported mode of familial communication, followed by text messages, Facebook, and e-mail. Most people reported weekly or more frequent contact with the relatives they felt closest to, and less frequent contact (from monthly to every few years) with other relatives. Most also reported open health information sharing between and among relatives, again varying with geographic distance and closeness of the relationship.

Six respondents named biological relatives who they are not in any contact with; three named brothers or cousins who they are out of contact with for relationship reasons (“there is a cousin that I’m not in touch with and would prefer not to be in touch with”); and three named cousins who live internationally where contact is difficult (“I can’t even remember the last time I saw him [cousin]. And it wasn’t because there was a rift... we just didn’t grow up together the way that the other, you know, cousins did.”)

### Risk notification plans

All participants said they would plan to share the results in the two scenarios with at least some of their relatives. Two people named individual relatives who they would not notify because of relationship issues (“My sister tells me only, and I tell her only, but otherwise we don’t share our medical situation with the other members of the family. … we have learned not to, you know, even if you have a hangnail, don’t tell [our mother] … she worries a fair amount.”

### Attitudes about KPWA involvement in risk notification

The majority of participants were open to all three types of KPWA involvement in familial risk notification for both result scenarios (Table 3). The scenario where KPWA contacts relatives directly had the most support, with 19 of the 20 participants indicating support. For KPWA contacting the relative’s KPWA physician, 17 people were supportive; one was not, and two were unsure. For KPWA placing an alert in relative’s medical record, 15 people were open; 3 were not, and 2 were unsure.

**Table 3:** Attitudes about KPWA involvement in risk notification

Outreach type Summary of findings	Quotes
KPWA contacts family members directly	
Open FBN1: 7 (40%) RYR1: 11 (55%)	“Oh, yeah. I think that would be wonderful ... it would be so much easier instead of me trying to spit out what I think they need to have done or whatever. You folks could do it in a more—they know what they are talking about and in a more business-like, you know, informational way than I could.” (FBN1)
	“You know, you guys are really good about identifying yourself, I think that would be fine.” (RYR1)
Open, with conditions FBN1 12 (60%) RYR1 8 (40%)	“Yes, provided I had the opportunity to alert them to that and get their permission … they would certainly have the right to refuse” (FBN1)
	“Yeah. Go ahead and they can contact the family member. I’d prefer to do it first, but it’s important enough that they should be contacted anyway.” (RYR1)
Not open to this* FBN1: 1(5%) RYR1: 1 (5%)	“No. I would do it ... they would be angry and upset and hurt if they did not get that information from me.”
	“Nope. That’s my job.” (RYR1)
Not sure: 0	N/A
KPWA contacts family member’s KPWA physician	
Open FBN1: 6 (30%) RYR1: 10 (50%)	“Absolutely ... I would think that that would be even more efficient than me sending a message that would not be very precise, you know, it would not fill in a lot of gaps. So it makes sense to have somebody with the data, the actual data provide the information.” (FBN1)
	“That’s, you know, I think—physician to physicians, fine.” (RYR1)
Open, with conditions FBN1: 11 (55%) RYR1: 8 (40%)	“I think I’d rather have that conversation with the family members first and see, you know, what their preferences were.” (FBN1)
	“I would say I would have to talk to them first and see if they would want their physicians to know what’s going on. I do not know why they would not, but still, I guess it’s a privacy thing.” (RYR1)
Not open to this FBN1: 2 (10%) RYR1: 2 (10%)	“No ... that one I do not want to cross I think. I do not want to cross that line.” (FBN1)
	“Yeah, not comfortable [with this].” (RYR1)
Not sure FBN1: 1 (5%) RYR1: 0	“I do not know. Maybe I would not—I am not sure. … Well, I really do not know.” (FBN1)
KPWA places notes in family member’s EMR	
Open FBN1: 5 (25%) RYR1: 8 (40%)	“Yeah. I think that’s a good thing. I really think that it’s important for people to know what sort of things they might be at risk for, since they have the technology to find out some things now. I think it’s a good thing.” (FBN1) “If I delay in talking with [my relatives], then that’s on me. But it’s more important the note be there then to have less worry. And there should be less worry anyway.” (RYR1)
	“In this case I think it’s a little more serious [than FBN1]. It seems like if you are likely to die—surgeries are more and more common. And it seems to me that [KPWA placing note in relatives’ EMR] that would be an appropriate thing to do.” (RYR1)
Open, with conditions FBN1: 10 (50%) RYR1: 9 (45%)	“I am fine with it. I probably would have to check with my siblings and make sure they are okay with it. I would not want them to be surprised or, you know, why was a note there and not know about it.” (FBN1)
	“I would talk to them first and say what do you think, if they can do that. Or can—I was going to say, or can the doctor give you a letter to put in your file.” (RYR1)
Not open to this FBN1: 3 (15%) RYR1: 3 (15%)	“No, I do not want to put things in other people’s medical records.” (FBN1) “And putting a note in the medical record, I would, you know, it’s a little—it’s—I would still not opt for that. I still think that would be risky in this uncertain political climate.” (RYR1)
Not sure FBN1: 2 (10%) RYR1:0	“I think the first thing to come to mind when you say that would be; would that, in any way, affect their access to insurance?” (FBN1)

*Single participant

Among participants who were supportive of direct outreach, the main motivation was to ensure that potential risk gets communicated to the relative, either so they would know or so they could take their own clinical action, which in most cases was viewed as getting testing (“I would want them to know so they can get checked”). One participant noted: “Genetic testing is hard to get, I discovered. My daughter went in and asked for it, and was told that they wouldn’t test her until I was tested.” Some participants noted that they felt that KPWA could relay all the necessary clinical information more accurately than they could themselves (“it makes sense to have somebody with the actual data provide the information”) or ensuring that their relatives are notified quickly (“I mean, if I haven’t told them within the week, I’d think there’s something wrong.”)

Some people who were supportive also indicated conditions or caveats. The primary caveat noted was the desire to either alert their relatives in advance or to get their relative’s permission before KPWA initiated direct outreach. Others, less commonly, expressed a desire to simply alert their relatives rather than to get permission (“I could give them a heads up that they would be getting a call”). Additional caveats included concerns about relatives’ privacy (“I wouldn’t just randomly give KPWA my family’s name and numbers without talking to them first”), and not wanting to negatively affect their own relationship with their relative. We also noted some concern about potential insurance discrimination, as in one participant who said: “I would want assurance that it would in no way jeopardize the ability [of my relative] to get health care, cost of health care and service.”

For people who were not supportive of any kind of KPWA involvement in risk notification, concerns were similar to the caveats listed by people who otherwise expressed support. These included concerns about the impact of notification on their relationship with the relative; the sentiment that risk notification was their own responsibility (“that’s my job”), the perspective that they were respecting their relatives’ choices (“my relatives are intelligent, independent, sentient, carbon-based beings. They have to decide for their own what they’re going to do with the information.”), or concerns about insurance discrimination (“I could affect my siblings’ future healthcare. Or [their] insurance, the premiums they pay, hmm. In the current political environment, no. Let’s not do that”).

### Attitudes on notifying children

In response to the question about whether their answers would depend on the age of the relative that might be notified, the primary response was that they would want to make sure the parents of the minor child be informed and part of that decision. One person stated, “[Parents] could decide whether they would like that information to be put in their children’s file. I don’t really have any responsibility, I mean, I can’t take on that responsibility of saying, oh, yeah, throw it in there.” Several people noted, however, that the clinical relevance of the result did not depend on age, and that they would want to make sure that the potential risk was communicated regardless of age.

### Consistency between scenarios

Half of participants saw no difference between the two result scenarios. For those who did differ in their responses between the two scenarios, all but one was more open to KPWA involvement in the malignant hyperthermia (*RYR1*) example. This was most often related to the clinical urgency associated with surgery in the *RYR1* example and the need to have that communicated. For example: “In [RYR1] I think it’s a little more serious. It seems like if you’re likely to die—surgeries are more and more common. And it seems to me that [KPWA placing note in relatives’ EMR] would be an appropriate thing to do.” Caveats or conditions were stipulated more often in the Marfan syndrome (FBN1) scenario, most typically a desire to alert relatives before health system contact was initiated, whereas in the *RYR1*, clinical scenario participants were less likely to note conditional support for direct contact.

### Relative identification

The 20 participants, in total, named 535 first- or second-degree relatives. Of those, 76 (14.2%) were identified by the participants as current or former KPWA members. After excluding 16 relatives listed as minors, as well as one relative defined as a step, rather than a biological relative, and two relatives for whom contact information was not provided, our analysis set included 57 potentially identifiable (hence potentially contactable) relatives.

Of these 57, 51 relatives (89.5%) were first-degree relatives (brother, sister, son, daughter, mother or father). First name, last name, and gender were reported for all relatives; birth date, month, year, and city/state were provided for 54 (94.7%). ZIP code and street address were the least often provided (40 and 44, respectively).

In total, we located a single match for 40 (70.2% of KP relatives; 7.5% of total identified) relatives within KPWA data. Of the 57 relatives, we located a single KPWA member match for 29 of them (50.9%) using name only (Table 4). Adding birth year or birth month resulted in a match with only one person in KPWA administrative data for an additional nine people (66.7%). Searching for last name and birthdate only resulted in an additional two relatives for which there was a single match in the KPWA system. Searching resulted in 2 to 11 KPWA member matches for an additional seven relatives (12.3%). For the remaining 10 relatives (17.5%), 29 or more matches resulted; mostly due to very common first or last names.

**Table 4:** Relative matching results

Total	57	100.0%
Single match of a KPWA member on last name + first name	29	50.9%
Additional single match adding birthyear	7	12.3%
Additional single match birthyear + birth month	2	3.5%
Single match on last name + birthdate only	2	3.5%
Total single matches	40	70.2%
Matched on 2–11 KPWA members	7	12.3%
Matched on 12 or more KPWA members (range 29–169, mean 137)*	10	17.5%

*All people who did not match last name + first name

## Discussion

We conducted a pilot study exploring attitudes about health system–led notification of familial genetic risk. We found support for three different types of direct involvement in risk notification, with the most unqualified support for health system outreach directly to relatives for a variant associated with higher clinical urgency (*RYR1*, malignant hyperthermia). Two other forms of outreach, either directly to a relative’s physician or by placing an alert in the relatives’ EMR, received slightly less unqualified support; the most common qualification was the desire to alert a relative to expect direct contact from the health system and/or get the relative’s permission for such outreach prior to a contact attempt. Results were similar across the sample, regardless of personal history of colorectal cancer.

We found an average of 2.9 relatives per proband who also received care in our health system, suggesting a potential opportunity to increase the uptake of genetic testing among eligible relatives through direct outreach. We could identify a single matching KPWA member using name and birthdate only for 70% of first- or second-degree relatives; we could narrow the pool to 10 or fewer KPWA members for an additional 20%. This suggests that the health system could identify relatives with a minimum of manual work in most cases; while in the remaining 10%, especially those with particularly common names, a more intensive identification process might be required.

Taken together, these results suggest that health system-led direct outreach to relatives is both feasible and acceptable to the index patient, with many people expressing relief at the idea of help with such outreach. The presence, even in our small sample, of estranged relatives that would go uncontacted is consistent with other literature and suggests a clear potential benefit for health system–led outreach in the form of increased referral and uptake of genetic testing among relatives. A survey of US-based genetic counselors found that 46% had encountered cases where a patient refused to inform a relative of their risk (Dugan et al. 2003). Contextual or situational factors, family relationships, and sociodemographic factors all influence whether patients will inform their relatives of a potential genetic predisposition (Wiens et al. 2013; Rodriguez et al. 2016; Gilbar 2007; Elrick et al. 2017). A systematic review of communication of genetic risk information within families found that physical or emotional distance from relatives, family communication patterns, or relationship issues, not feeling prepared, fear of worrying or distressing relatives, or decisions that the relative will not benefit from the information are all barriers to informing relatives (Wiseman et al. 2010). Together with the low uptake rates of familial testing, these studies suggest clear limitations of an index-patient led risk notification process and a clinical rationale for pursuing direct outreach to relatives.

The US would by no means be the first country to explore direct outreach to relatives. Family-based approaches to genetic risk disclosure and cascade screening are being planned or implemented in Australia (Forbes Shepherd et al. 2017; Watts et al. 2011), France (D’Audiffret Van Haecke and de Montgolfier 2016), and UK (Dheensa et al. 2017; Mitchell et al. 2016). Early work in familial hypercholesterolemia cascade screening in the Netherlands found that being directly approached by the program for testing was broadly acceptable to relatives and resulted in high acceptance of screening (van Maarle et al. 2001). An Australian study of the impact of direct outreach to relatives found that at 2 years of follow-up, 40% of relatives who had received direct outreach had received genetic testing and had their genetic status defined compared to 23% in the no-direct outreach comparison group (*p* < 0.001). No complaints were received about privacy concerns in that study (Suthers et al. 2006). Other research exploring direct outreach to relatives has found similar results and acceptability levels among patients in Finland (Aktan-Collan et al. 2007) and the UK (Hadfield et al. 2009).

To date, our study represents some of the earliest work exploring the acceptability of system-based approaches to familial risk notification in the more fragmented US healthcare system, with most US studies focused on ways to support index patients with informing their family members (Samimi et al. 2017; de Geus et al. 2015).

The health professional duty to family members in the context of genetic testing remains controversial in the USA. The sacrosanct nature of the physician-patient relationship is a widely accepted ethical foundation of medical practice, built on the concept of patient autonomy and respect for the individual right to privacy. However, since the completion of the Human Genome Project, this concept has been challenged in the context of genetic testing, in which shared genetic information and clinical implications for family members are the norm (Offit et al. 2004; Burke and Press 2006). In cross-sectional and qualitative US-based studies, patients have reported conceptualizing genetic information as familial, not individual, and expressed willingness to have providers notify relatives if the patient does not do so (Dheensa et al. 2016a; Pentz et al. 2005; Kohut et al. 2007; Plantinga et al. 2003; Benkendorf et al. 1997).

Thus, several scholars have suggested ethical rationale for expanding the health professional role to include direct communication of genetic risk to relatives, based either in a solidarity-informed ethic of care that includes all biological relatives (Weaver 2016); a “family covenant” that recognizes a physician’s duty to all family members in which all family members would participate voluntarily (Doukas and Berg 2001); or the idea of a “joint account” that emphasizes the unique nature of shared genetic information among family members (Parker and Lucassen 2004). Of course, these models may create tension with a relative’s right “not to know” their genetic risk (Juth 2014) and with the prevailing reliance on individual patient privacy and autonomy. Case law has been limited about the physician duty to warn relatives (Clayton 2000; Gilbar and Foster 2016); a recent review has concluded that healthcare providers are neither required nor permitted to warn at-risk relatives without the consent of their patients (Rothstein 2018).

Despite legal protections that relieve them of an obligation to contact relatives directly, clinicians may retain a sense of duty to their patients’ relatives. A survey of US genetic counselors found that 63% agreed that they feel an obligation to inform at-risk relatives (Dugan et al. 2003). A study of medical geneticists found that 69% reported an obligation to notify their patients’ relatives of a potential genetic risk (Falk et al. 2003). However, in qualitative studies, physicians often express concerns about the ethical tensions between individual and family interests and interpretation of duty (D’Audiffret Van Haecke and de Montgolfier 2016; Dheensa et al. 2017; Dheensa et al. 2016b).

We acknowledge several limitations. This was a pilot study in a single integrated system, with a relatively homogenous sample of people who had previously consented to participation in genetic research and who agreed to provide contact information for their relatives. How other patients in our healthcare system, or in other healthcare systems, would respond to the scenarios should be confirmed with additional research. Further, because these individuals had agreed to genetic testing but had not yet received results, our interview guide centered on hypothetical scenarios only. Responses may not translate to actual preferences and/or behavior in the face of actual test results, including secondary findings not anticipated at the time of study enrollment. Our use cases were designed to elicit preferences and concerns surrounding three types of health system involvement, not to simulate a complete informed consent process or describe in detail the potential risks and benefits of testing for each variants. We did not probe for scenarios in which the patient died before receiving results, nor did we probe specifically on a relative’s right not to know genetic information or explore the logistics of how such programs might work in practice or for relatives outside of our system. Further research can explore these ideas more fully. Furthermore, our identification efforts were passive (i.e., we did not directly confirm that the individuals we identified in our records were the biological relative identified by the index participant), so some misclassification may remain.

Our work provides early evidence that health system involvement in familial risk notification is acceptable to patients in a US integrated health system. Future research is needed to understand relatives’ attitudes about being directly contacted, the attitudes and concerns of people in other integrated systems, as well as in more ethnically and economically diverse populations, and how precisely to design and implement direct outreach processes while insuring privacy protection. However, ethically designed and implemented familial notification systems in a US setting could positively impact the care and health outcomes of people from families at risk of genetic disease.

## Data Availability

The data from this study are qualitative in nature and are retained at Kaiser Permanente Washington Health Research Institute. The datasets used and/or analyzed during the current study are available from the corresponding author on reasonable request.

## Abbreviations

ACMG: The American College of Medical Genetics and Genomics
CRC: Colorectal polyps
DNA: Deoxyribonucleic Acid
EMR: Electronic Medical Record
HIPAA: Health Insurance Portability and Accountability Act
KPWA: Kaiser Permanente Washington
KPWHRI: Kaiser Permanente Washington Health Research Institute
NWIGM: The Northwest Institute for Genomic Medicine Biorepository
UW: The University of Washington
